# Monitoring the Influence of Low CVP Versus Stroke Volume-Guided Fluid Therapy on Sublingual and Intestinal Microcirculatory Perfusion

**DOI:** 10.1213/ANE.0000000000007734

**Published:** 2025-09-23

**Authors:** Zühre Uz, Iris M. Jongerius, Denise P. Veelo, Bülent Ergin, Thomas M. van Gulik, Can Ince, Collaborators, Matthias P. Hilty

**Affiliations:** From the 1 Department of Surgery, Amsterdam UMC, University of Amsterdam, Cancer Center Amsterdam Meibergdreef 9, Amsterdam, the Netherlands; 2 Department of Intensive Care, Laboratory of Translational Intensive Care, Erasmus MC, University Medical Center Rotterdam, Rotterdam, the Netherlands; 3 Department of Anesthesiology, Amsterdam UMC, University of Amsterdam, Meibergdreef 9, Amsterdam, the Netherlands.

The primary goal of fluid therapy is to optimize tissue and microcirculatory perfusion.^1,2^ Inadequate or excessive administration may lead to hypoperfusion or fluid overload, and result in adverse outcomes.^2,3^

While conventional hemodynamic targets focus on systemic variables such as mean arterial pressure (MAP)—recent studies suggest that specific MAP thresholds may not reflect adequate microcirculatory perfusion. A direct assessment of microcirculatory tissue perfusion may help clinicians tailor hemodynamic management and intravenous fluid therapy more effectively.^3,4^

To address this question, we assessed the relationship between fluid management and microcirculatory adequacy in a substudy of the GALILEO trial—a randomized controlled trial (RCT) comparing a low central venous pressure (Low-CVP) strategy with stroke volume-directed fluid therapy (SVDFT) during major liver surgery.^5^ The original trial found no differences in blood loss or clinical outcomes between strategies.

This substudy also evaluated the feasibility of intraoperative sublingual and intestinal microcirculatory monitoring using handheld vital microscopy (HVM) under these two fluid management strategies.

## METHODS

The GALILEO trial was a single-center, surgeon and patient-blinded RCT.^5^ This study was approved by the Medical Ethics Committee (MEC) of the Amsterdam UMC, location AMC (MEC number: MEC2016_004; February 24, 2016) and was registered (Netherlands Trial Register, NTR5821). Written informed consent was obtained from all subjects.

See Supplemental Digital Content 1, Supplemental Material 1, https://links.lww.com/AA/F495 for standardized perioperative anesthetic care applied in both groups.

Both groups received background fluid of 0.5 mL/kg/hour with crystalloids. Positive-end-expiratory pressure was set to 0 to avoid possible effects on the CVP. Blood transfusions followed institutional protocols. Patients were randomized to either stroke volume (SV)-directed fluid therapy (SVDFT group) or low CVP management.

### Low-CVP Group

The maximal allowed CVP was 5 mm Hg. Before incision, all patients in the low-CVP group received a Furosemide bolus (0.5 mg/kg). If the CVP rose >5 mm Hg during surgery, a nitroglycerin (NTG) infusion was started at (0.05–0.2 μg/kg/min). If NTG was contraindicated due to hypotension or did not lower CVP, the patient was placed in 15º head-up (reverse-Trendelenburg) position. Additional fluids were only allowed during ongoing bleeding or when vasopressor use was unacceptably high. After the resection phase was complete, intravascular volume resuscitation was permitted.

### Stroke Volume (SV)-Directed (SVDFT) Group

MAP target ≥ 60 mm Hg or ≤ 30% below baseline in hypertensive patients. Patients received fluid bolus after the induction phase, according to a goal-directed fluid therapy (GDFT) protocol focusing on SV, see Supplemental Digital Content 2, Supplemental Material 2, https://links.lww.com/AA/F496. Briefly, optimal SV was identified based on the response (increase, no effect, or decrease of SV) to the fluid bolus after induction. The SV-triggered fluid bolus management was continued until the patient was discharged to the nursery ward.

### Microcirculatory Monitoring

Incident darkfield imaging (IDF) embedded in an HVM was used to assess sublingual and intestinal serosal microcirculation.^6^ Sublingual and intestinal measurements were made after skin incision (T0) and just before skin closure (T1). Only sublingual microcirculation was measured 24 hours after surgery. Images were analyzed by clinically validated automated microcirculation analysis software called MicroTools.^7^ Baseline characteristics, intraoperative parameters, and clinical outcomes were also collected.

## RESULTS

The original GALILEO trial enrolled 40 patients. In this substudy, 38 patients were included for microcirculation measurement (N = 18 in the SVDFT group and N = 20 in the Low-CVP group). The two groups did not differ in baseline characteristics (see the Table for baseline, intraoperative, and postoperative characteristics). NTG was used in 7 patients to reduce CVP. In 4 patients, reverse Trendelenburg was used; 2 of these patients had already received NTG.

**Table.** Baseline Characteristics, Intraoperative, and Postoperative Parameters

Baseline characteristics	SVDFT (n = 18)	Low-CVP (n = 20)	
Male, n (%)	9 (50%)	12 (60%)	
Age, median (IQR), y	66 [58–68]	66 [54–74]	
BMI, median (IQR), kg⋅m^–2^	26 [23–27]	24 [22–28]	
ASA score, n (%) I II III IV	1 (6%) 13 (72%) 4 (22%) 0	2 (10%) 11 (55%) 6 (30%) 1 (5%)	
Localization of liver resection, n (%) Left hemihepatectomy Right hemihepatectomy Minor resection	5 (28%) 10 (55%) 3 (17%)	9 (45%) 9 (45%) 2 (10%)	
Portal vein embolization, n (%)	7 (39%)	3 (15%)	
Pathology malignant, n (%)	17 (94%)	16 (80%)	
Pathology nonmalignant, n (%)	1 (6%)	4 (20%)	
Intraoperative			*P* value
Blood loss, median (IQR), mL	1275 [772–1713]	1425 [854–1952]	.55
CVP during transection, mean (SD), mm Hg	7 (3)	3 (2)	<.01
Fluid balance at start transection phase, mean (SD), mL	180 (800)	−730 (430)	.04
Fluid balance end of surgery, mean (SD), mL	+620 (760)	−200 (560)	<.01
Noradrenaline use, median (IQR), μg·kg^–1^·min^–1^	0.02 [0.02–0.05]	0.05 [0.04–0.10]	.02
Cardiac Index, mean (SD), L·min·m^–2^	2.58 (0.43)	2.34 (0.48)	.10
Urine output total, median (IQR), mL	589 [330–1010]	1280 [850–1410]	<.01
Need for blood transfusion, n (%)	0 (%)	4 (20%)	.04
Colloids administered, median (IQR), mL	500 [0–625]	500 [100–1000]	.36
Crystalloids administered, median (IQR), mL	2350 [1530–2875]	1800 [1365–2150]	.10
Highest lactate, mean (SD), mmol·L^–1^	3.8 (1.2)	3.0 (1.3)	.06
Postoperative			
Fluids administered 0–12 h postoperative, median (IQR), mL	2110 [1390–3040]	1490 [1240–2940]	.58
Urine output 0–12 h postoperative, mean (SD), mL	560 (270)	640 (340)	.41
Need for blood transfusion, n (%)	1 (6 %)	4 (20%)	.38
Highest lactate^a^, median (IQR), mmol·L^–1^	4.5 [3.4–5.6]	4.3 [3.4–5.5]	.61
Clavien Dindo Score at 90 days ≥ 3, n (%)	7 (39%)	10 (50%)	.53

Abbreviations: ASA, American Society of Anesthesiologists; BMI, body mass index.

a Data collected during the first 5 days postoperative.

Total perioperative fluid administration did not differ between groups. The SVDFT group had a more positive intraoperative fluid balance than the Low-CVP group (P < .01), consistent with the Low-CVP group receiving diuretics.

**Figure.**
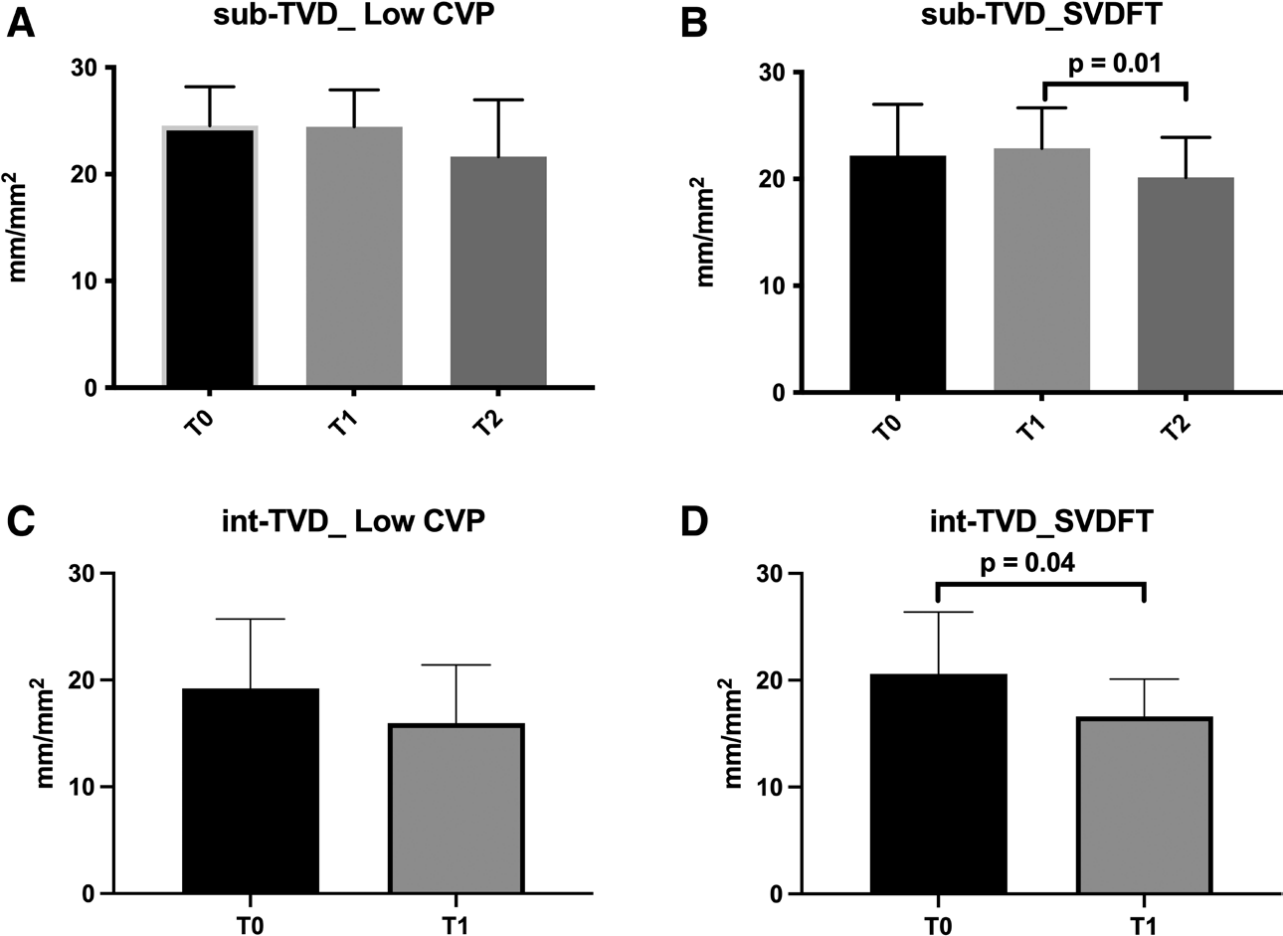
Total vascular density (TVD; mm/mm^2^) in the intestinal and sublingual microcirculation: low-CVP vs SVDFT. A, No significant changes of TVD in the low-CVP sublingual microcirculation. B, Significant decrease in the TVD between T2 and T1 in the SVDFT sublingual microcirculation. TVD (mean ± SD): T2: 20.1 ± 3.7 mm/mm² vs T1: 22.8 ± 3.1 mm/mm², *P* = .01. C, No significant changes of total vascular density (TVD) in the low-CVP intestinal microcirculation. D, Significant changes of TVD in the SVDFT intestinal microcirculation. TVD (mean ± SD): T0: 20.6 ± 5.8 mm/mm² vs T1: 16.6 ± 3.5 mm/mm², *P* = .04. int-TVD, intestinal total vessel density; low-CVP, low central venous pressure; sub-TVD, sublingual total vessel density; SVDFT, stroke volume directed fluid therapy; T0, after skin incision; T1, after skin closure; T2, 24 hours postoperative.

The intestinal total vessel density (TVD, mm/mm^2^) in the SVDFT group decreased at the end of surgery (T1; *P* = .04), as did the TVD in the sublingual microcirculation 24 hours after surgery (T2; *P* = .01) (Figure). In contrast, the sublingual and intestinal microcirculation in the Low-CVP group, remained at baseline for the whole perioperative phase. No differences in the convective component of microcirculatory perfusion (RBC velocity) were found between SVDFT and the Low-CVP groups. As with the main GALILEO trial result, clinical outcomes did not differ between the low CVP and SVDFT groups.

## DISCUSSION

Tissue perfusion and oxygenation are central objectives in perioperative fluid management.¹,³ This feasibility study found that TVD—an indicator of microcirculatory oxygen delivery capacity—decreased in the SVDFT group but not in the Low-CVP group. Convective parameters were similar between groups.

Our microcirculatory findings may be due to the greater positive fluid balance in the SVDFT group, possibly leading to interstitial edema, capillary compression, and reduced microvascular density.² Such a possibility would align with previous studies where TVD was reduced by fluid overload but could be reversed with diuretic therapy.^8,9^

The relationship between NTG and reverse Trendelenburg in the Low-CVP group may also have played a role in preserving TVD by enhancing perfusion pressure and promoting microcirculatory recruitment. A concept supported by a 2009 study finding similar improvement with NTG in heart failure patients.^10^ Future studies are needed to clarify the relationship between NTG and diuretic use and microcirculatory adequacy.

Our study has limitations. Two patients were excluded from the SVDFT group in the original GALILEO trial due to lack of informed consent (1) and technical issues with microcirculation monitoring (2).

In addition, our study focused on liver resection surgery, and our results thus may not be generalizable to other surgical procedures. Finally, our overall sample size is small, limiting our ability to draw conclusions from our findings. Nonetheless, the study demonstrates the feasibility of intraoperative microcirculatory monitoring at two different organs during different hemodynamic management modalities, and supports further research into optimizing perfusion-based fluid management.

## ACKNOWLEDGMENTS

The authors thank Mathias Hilty, Lucinda Shen, and Timothy Mungroop who were indispensable in the completion of this study. Professor Thomas M. van Gulik and Professor Can Ince do share the last authorship.

## DISCLOSURES

**Conflicts of Interest:** None. **Funding:** None. **This manuscript was handled by:** Avery Tung, MD, FCCM.

## Supplementary Material
