# Supplementary material for: Monitoring the Influence of Low CVP Versus Stroke Volume-Guided Fluid Therapy on Sublingual and Intestinal Microcirculatory Perfusion
Source: Anesth Analg. 2025 Sep 23;142(3):613–6. doi: 10.1213/ANE.0000000000007734 (PMC12871401; doi:10.1213/ANE.0000000000007734)
Supplement: Supplementary file 1 [file ane-142-613-s001.pdf]

Supplemental Content 1. Standardized Perioperative Anesthetic Care

1. **Preoperative preparations**

Patients were admitted one day prior to surgery and received standard of care.

2. **Induction of anesthesia**

In the operating room, general anesthesia was induced with propofol (2-3 mg·kg^{-1}), sufentanil (0.2-0.6 mg·kg^{-1}) and 1 mg·kg^{-1} rocuronium to facilitate intubation of the trachea. A bolus of 0.25 mg·kg^{-1} esketamine was given. An arterial catheter (right or left radial artery), a right jugular vein tri-lumen central catheter, a double lumen gastric cannula and urinary catheter (suprapubic in men) were inserted. Cefazoline (1000 mg) and Metronidazol (500 mg) were given intravenously prior to incision. Cefazoline and Metronidazol were repeated according to the national antibiotic guidelines.

3. **Maintenance of anesthesia**

The lungs were mechanically ventilated with pressure regulated volume control with a volume of 6 ml·kg^{-1} using ideal body weight. No positive end-expiratory pressure (PEEP) was used as this can affect the CVP. After induction general anesthesia was maintained with sevoflurane at a minimal alveolar concentration of 1 and sufentanil continuously at the rate of 0.5 -1 µg·kg^{-1}·hr^{-1}. After incision a bolus of local anesthetics was given around the wound. At the end of surgery wound catheters were placed by the surgeon as previously described by Mungroop et al.[17] and a second bolus of local anesthetics was given after placement.

4. **Postoperative Care**

The endotracheal tube was removed in the operating room and patients were discharged to the recovery room. Pain was treated with acetominophen, an NSAID, continuous infusion of bupivacaine 0.125% at 12 ml·hr^{-1} given over the wound catheters and a Patient Controlled Analgesia pump with morphine (or buprenorphine if morphine was contraindicated).
