# Supplementary figures and images for: Monitoring the Influence of Low CVP Versus Stroke Volume-Guided Fluid Therapy on Sublingual and Intestinal Microcirculatory Perfusion
Source: Anesth Analg. 2025 Sep 23;142(3):613–6. doi: 10.1213/ANE.0000000000007734 (PMC12871401; doi:10.1213/ANE.0000000000007734)

Supplemental figure 1: Perioperative Stroke Volume Directed Fluid Therapy Protocol

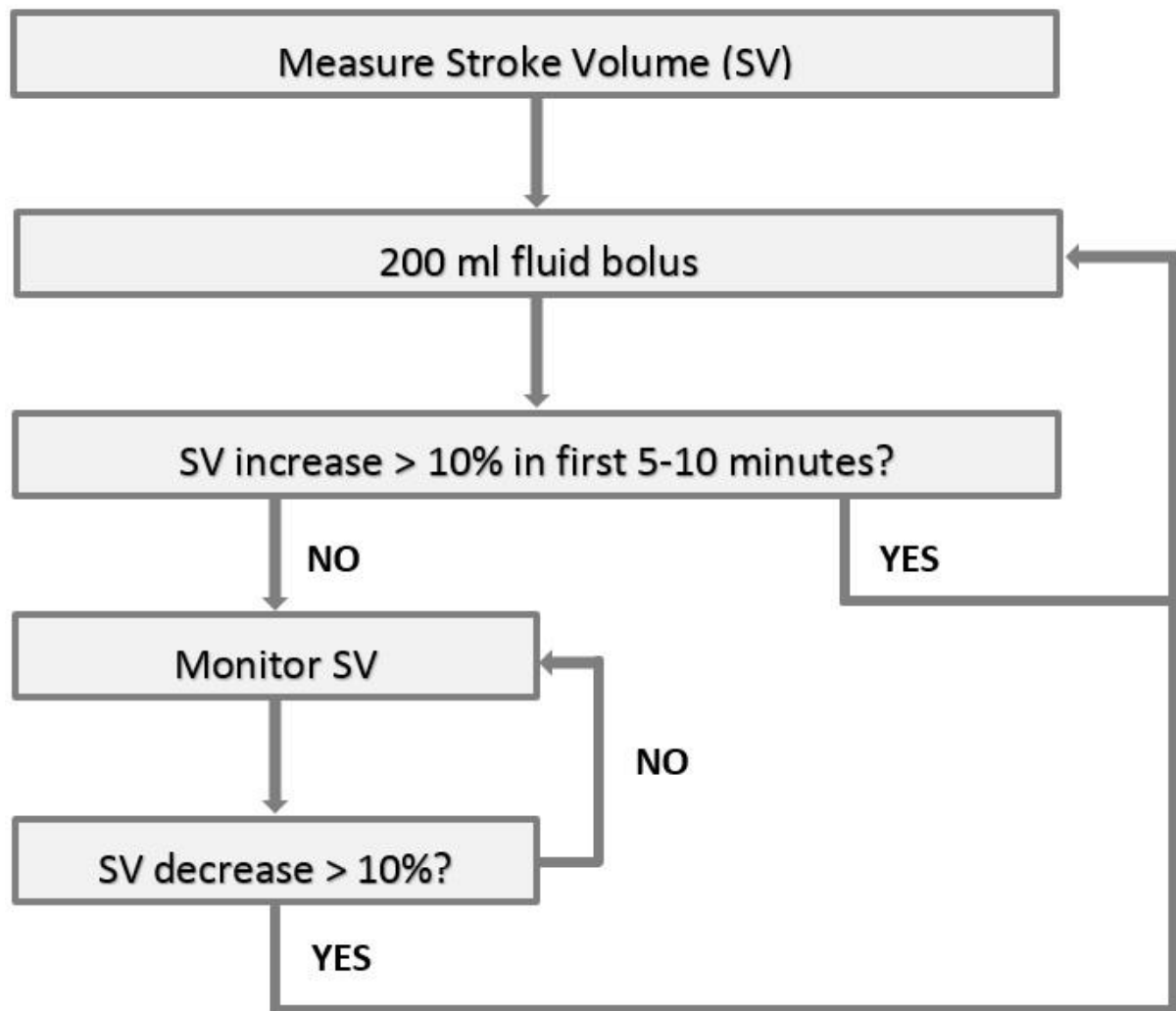

Supplement: Supplementary file 2 [file ane-142-613-s002.pdf]
